# A programme theory for liaison mental health services in England

**DOI:** 10.1186/s12913-018-3539-2

**Published:** 2018-09-27

**Authors:** Allan House, Elspeth Guthrie, Andrew Walker, Jenny Hewsion, Peter Trigwell, Cathy Brennan, Mike Crawford, Carolyn Czoski Murray, Matt Fossey, Claire Hulme, Adam Martin, Alan Quirk, Sandy Tubeuf

**Affiliations:** 1 Leeds Institute of Health Sciences, University of Leeds, Leeds, UK; 2 Clinical Research Network National Coordinating Centre, National Institute of Health Research Clinical Research Network, Leeds, UK; 3 National Inpatient Centre for Psychological Medicine, Leeds and York Partnership NHS Foundation Trust, Leeds, UK; 4 Faculty of Medicine, Department of Medicine, Imperial College, London, UK; 5 Veterans and Families Institute for Military Research, Faculty of Health, Social Care and Education, Anglia Ruskin University, Chelmsford, UK; 6 Academic Unit of Health Economics, Leeds Institute of Health Sciences, University of Leeds, Leeds, UK; 7 College Centre for Quality Improvement, Royal College of Psychiatrists, London, UK

**Keywords:** Consultation-liaison psychiatry, Liaison mental health services, Theories of change, Logic models, Programme theory

## Abstract

**Background:**

Mechanisms by which liaison mental health services (LMHS) may bring about improved patient and organisational outcomes are poorly understood. A small number of logic models have been developed, but they fail to capture the complexity of clinical practice.

**Method:**

We synthesised data from a variety of sources including a large national survey, 73 in-depth interviews with acute and liaison staff working in hospitals with different types of liaison mental health services, and relevant local, national and international literature. We generated logic models for two common performance indicators used to assess organisational outcomes for LMHS: response times in the emergency department and hospital length of stay for people with mental health problems.

**Results:**

We identified 8 areas of complexity that influence performance, and 6 trade-offs which drove the models in different directions depending upon the balance of the trade-off. The logic models we developed could only be captured by consideration of more than one pass through the system, the complexity in which they operated, and the trade-offs that occurred.

**Conclusions:**

Our findings are important for commissioners of liaison services. Reliance on simple target setting may result in services that are unbalanced and not patient-centred. Targets need to be reviewed on a regular basis, together with other data that reflect the wider impact of the service, and any external changes in the system that affect the performance of LMHS, which are beyond their control.

## Background

Liaison mental health services (LMHS) in England have become a focus of the Department of Health’s plans to develop key areas of mental health provision [1]. LMHS provide assessment and treatment for people with physical and mental health problems in the acute hospital setting, and treatment and management of people with complex physical and mental health problems in primary and secondary care. LMHS have the potential to improve both the quality of care and overall outcomes for people with mental and physical health problems [2], and there is also an expectation that liaison services in the acute hospital setting will produce cost savings by reducing length of stay [3, 4].

Liaison services should reflect to a certain degree the size and acuity of the acute hospital they serve, but there is great heterogeneity in the composition, purpose, size and activity of liaison services [5], which is not fully explained by hospital variation.

There is some evidence that LMHS may lead to a variety of improved outcomes including; reduction in length of stay [2, 6, 7]; reduced wait times in ED [8]; and high levels of patient and referrer satisfaction [9, 10] but the mechanism and process by which liaison services might produce these changes is for the most part unclear. Programme theories explain how interventions or services are understood to contribute to intended outputs and outcomes. They are useful ways to bring together existing evidence about a service, clarifying where there is agreement and disagreement about how the service is understood to work and where there are gaps in the evidence [11].

Logic models are widely accepted tools, used to aid programme evaluation and to develop programme theory. They present in a diagrammatic form the relations between available resources or inputs, planned activities, outputs and desired outcomes and impact [12]. Logic modelling has been used previously in liaison mental health settings to aid the re-organisation of a psycho-oncology service [13], and to integrate mental health into a programme for the prevention and health promotion in chronic physical disease [14]. More recently, the Department of Health and Social Care in England has produced a logic model for urgent and acute LMHS in support of its plans to expand provision for LMHS on a nationwide basis [1].

These models describing LMHS are all relatively simple and postulate direct linear paths from resources to activities to outputs to outcomes and then impact. Such models struggle to capture the complexity of liaison services, and particularly the NHS England model [1] simplifies and overstates the causal contribution of liaison services to intended outcomes (e.g. reduction in length of stay or improved patient outcomes). The heterogeneity of liaison services both in terms of composition and function suggests that they are better represented by complicated logic models, which have both simultaneous and alternate causal strands, operating in different contexts [11]. In addition, most logic models show a ‘one pass’ through the intervention and depict services in isolation rather than as part of an established system, which is constantly in flux. Thus, many service-level interventions depend on or lead to unintended consequences within the service or changes in other organisational components not initially envisaged. For example, previous work has shown increases in the number of referrals [15], referral patterns and type of interventions [16] after the introduction of new services or expansions in existing liaison services. As services develop, and encounter unintended consequences, they naturally adapt to attempt to accommodate, or unanticipated strains are encountered in other parts of the system.

In this paper, we build on previous work evaluating liaison services in England, to develop a programme theory for LMHS in the acute hospital setting. Our objectives were to identify:

- relevant inputs, activities, outputs and outcomes regarding liaison services which could be used to develop theories of how liaison services may lead to improved outcomes and
- different dimensions of complexity which may shape or hinder the application of these theories to different components of liaison services.

## Methods

This work formed part of the first phase of a programme funded through the National Institute for Health Research to evaluate the cost-effectiveness and efficiency of different configurations of LMHS in England (Liaison Psychiatry: Measurement and Evaluation of Service Types, Referral Patterns and Outcomes [LP-MAESTRO] 13/58/08) [6]. Ethics approval was received from North of Scotland Research Service (REC reference: 15/NS/0025) and NHS Trust approvals were obtained. All participants provided informed written consent. This paper synthesises three sources of data collected in relation to the above programme and includes:

- Data from a nationwide survey of all 168 acute hospitals in England which have a LMHS [17].
- Qualitative interviews with 73 liaison and acute hospital staff who worked in hospitals with liaison services that were representative of different models of service delivery
- Relevant literature, and reports regarding LMHS provision in England, including historical accounts of LMHS [18, 19] evaluation of liaison services [2, 3, 6, 7, 20–28] and key national reports [29–36].

### Nationwide survey

The main data from the nationwide survey are published separately [17]. In summary, all 168 acute hospitals in England with a LMHS were sent a questionnaire enquiring about the size, configuration, operational function and treatment/assessment focus of each liaison service. Acute hospitals were defined as hospitals with an emergency department (ED), and there are 179 in total in England, with 11 hospitals without any liaison service at all. The response rate to the survey from the 168 hospitals was 100%, due to professional networking by members of the Faculty of Liaison Psychiatry, Royal College of Psychiatrists, and a recognition by professional colleagues that the data were necessary to underpin any potential expansion in liaison services in England. Hospital services that responded to the survey were clustered using a latent class model [37]. This method enabled classification of service type without recourse to any pre-conceived ideas or preconceptions of what liaison services should be like, and suggested four patterns of service delivery depending upon size of service, salience of acute work, provision of outpatient clinics and differentiation of an old age – specific component of the service (see below) [38].

### Qualitative interviews

Seventy three individual in-depth, qualitative interviews were conducted with staff from 11 different hospitals chosen to represent hospitals with the 4 different types of liaison service. As services had diverse configurations and sizes, there was a significant range in the number of participants interviewed from each service, so between 4 and 11 interviews were conducted at each hospital. Liaison staff (i.e. mental health practitioners working in liaison mental health services) and acute hospital staff, who had experience of referring to liaison LMHS, were included in the interview sampling. The methods are described in more detail in a separate paper, which focuses upon the barriers to integration of liaison services into acute hospital services (Keeble J, Walker A^,^ Guthrie E^,^ Trigwell P^,^ Quirk A^,^ Hewison J, House A. Integrated liaison psychiatry services in England: a qualitative study of the views of liaison practitioners and acute hospital staffs from four distinctly different kinds of liaison service. Submitted to BMC Health Services Research).

For the purposes of this paper, we focus upon the findings from five key topic areas: who are liaison services for?; what are liaison services? (inputs in the model); what do liaison services do and how do they affect change according to staff who work in liaison services? (activities in the model); what are the key patient and organisational outputs and outcomes?; what factors influence the context in which services operate?

## Findings

### Who are liaison services for?

There was a consensus amongst liaison staff that liaison services should primarily be for people with mental health problems who are currently in the acute hospital setting.

Most services did not have clear criteria or thresholds for referrals, and most teams offered assessment without judgments as to suitability. Several common clinical scenarios were described: self-harm; delirium and dementia in people with physical health problems; severe mental illness co-existing with physical health problems; mental health problems arising as a consequence of long-term physical illness or its medical treatment; physical illness exacerbated or caused by mental health problems - for example through poor adherence to treatment; people with unexplained persistent physical health symptoms, the severity and chronicity of which were disproportionate to suspected underlying disease mechanisms; people who experienced physical or psychological consequences of alcohol or drug misuse.

Many liaison staff described their service as being under-resourced and unable to cope with fluctuating demands from the acute hospital. There was also a fear of increased demand from the acute hospital, with a recognition that improved detection and recognition of mental health problems by acute hospital staff may result in significantly more patients being referred.

### Inputs: What are liaison services?

#### Survey Data

From the nationwide survey and cluster analysis, we identified 4 different kinds of liaison services, according to size, staffing, hours of service, components and function. These service configurations did not map onto published descriptions of liaison services [7] or commissioning guidelines from the Department of Health and Social Care [30]. They were in brief:

- A: small services, which in the main did not provide 24/7 cover or out-patient services (*n* = 46);
- B: services that provided 24/7 acute cover but very little non-acute work (*n* = 35);
- C: services that covered the acute work but offered non-acute care (*n* = 43);
- D: services that were less focused on the acute care pathway, provided non-acute care and had separate adults of working age and older adult teams (*n* = 44).

#### Interview data

There was great diversity in how services were configured, their clinical priorities and their historical development, but most were staffed by mental health nurses and liaison psychiatrists. Separate clinical or health psychology services existed in some hospitals and were often linked to specific units (e.g. renal or diabetic services). These psychology services operated entirely separately from liaison services offering brief non-acute psychological treatment, only linking with liaison or acute psychiatry if there was a concern about risk.

All liaison teams were supported by acute on-call psychiatry teams, which provided telephone advice and face to face assessments with patients if cases were considered complex, high risk or requiring assessment under the Mental Health Act.

Small teams were usually staffed by liaison mental health nurses with or without sessional input from a consultant liaison psychiatrist. Larger teams (20+ members) consisted of nurses and psychiatrists. Assessments were often carried out initially by nurses, and then re-assessed by a psychiatrist if necessary. Psychiatrists also tended to take the more complex referrals or those involving some aspect of pharmacological drug management. Some team members had areas of expertise (e.g. older adults) and worked predominantly or exclusively in these areas.

There was a balance between diversity and continuity of working either based on individual preference or on service needs, or a mix of both, and was aimed at balancing some need for special interest and experience, against the need for breadth of competence and some shared sense of priorities and working practices.

Some larger services provided out-patient treatment for patients, either originally seen as an in-patient or for patients referred by GPs or hospital consultants. A number of services also ran specialist out-patient clinics focused on specific problem areas (e.g. medically unexplained symptoms). There was a trade-off between the amount of out-patient work that could be undertaken and the ability to respond to acute referrals, 24 h a day.

### Activities: What do liaison services do and how do they affect change in patient and organisational outcomes?

#### Assessment

Teams described an interactive, patient-focused approach to assessment, which involved a detailed dynamic formulation of the patient’s mental and physical problems. The assessment usually lasted 60 min, following which the practitioner would discuss with the patient a plan. This could involve taking over the patient’s care and recommending transfer to a mental health bed, providing shared care with the medical/surgical team whilst the patient was in hospital, or providing advice to the medical team with signposting or referral to a community service post discharge.

All teams offered medical advice and co-management in certain cases e.g. management of agitated delirium, drug treatment for psychosis, and alcohol or drug withdrawal. Teams that had psychologists, therapists or mental health nurses trained in specific interventions (like cognitive behavioural therapy) offered brief interventions while patients were in the acute hospital, or a follow-up appointment after they had been discharged.

#### Shared working

This involved working collaboratively with acute hospital teams to implement agreed patient-care plans, which involve ongoing joint care. There was a tension between a desire to take over the care of the patient entirely, so that all recommendations were carried out, versus working in a more integrated way and having less direct influence over care.

Advice and guidance about the use of psychotropic medication in physically unwell patients was provided by liaison services, principally by liaison psychiatrists. In many acute hospitals, liaison professionals were not able to prescribe medications, as they were employed by a different organisation (i.e. a mental health trust), but they were able to give appropriate advice to medical and surgical colleagues.

#### Advice and intervention involving the law

Mental Health Act (MHA) work formed a key part of a consultant liaison psychiatrist’s role, particularly in settings with high numbers of MHA assessment referrals. If the patient remained on the acute ward following the assessment, the consultant liaison psychiatrist would usually be designated the Responsible Officer for the care of the patient under the MHA, which required on-going regular review to ensure the patient was being managed in a safe environment and being cared for appropriately. Liaison services also provided expert advice on complex cases requiring assessment under the Mental Capacity Act.

#### Change

Change therefore resulted from a formulation of the patient’s problems, which included an understanding of the interplay between physical and mental health, as well as the environment in which the patient was being managed. The mechanism of change was brought about by a variety of different activities which could include; direct action on the patient (psychological treatment or medication), changing the behaviour of clinicians involved in the care of the patient (implementation of fluid balance chart, or increasing levels of observation), or some form of environmental change (e.g. moving the patient to a side room to reduce the level of stimulation).

### What are the outcomes?

Staff described two main outcome groups: clinical and organisational. In EDs and acute wards an important clinical outcome for liaison professionals was preventing suicides in people they saw in suicidal crisis who had either attempted suicide or had strong suicidal thoughts or plans. The detailed assessment, careful management of risk and organised aftercare meant these patients were discharged safely or admitted to a psychiatric bed or managed safely on a medical or surgical ward whilst receiving life-saving physical intervention. Liaison professionals strongly valued this aspect of their role and perceived it as important to help people at their most vulnerable*.*

Other important clinical outcomes for liaison staff in all settings were: symptomatic improvement in patients’ mental health, prevention of a deterioration in mental health in patients with severe mental health problems, aiding physical recovery through addressing a new or historic mental health problem (e.g. depression in stroke patients), improving quality of life for people with complex, unexplained physical symptoms or long-term mental health problems, and improving adherence to medication for physical health problems (e.g. HIV anti-retroviral regimes).

There were several organisation targets which included: meeting specified response times, reducing length of stay in acute hospitals, reducing re-admission rates, compliance with 4 h wait targets in ED, and reducing frequent attendance at ED. There was a balance between the ability to respond quickly, the volume of patients who could be seen and the intensity of the intervention that could be provided. This tension between clinical outcomes and the setting of arbitrary organisational targets is discussed more fully below. For smaller teams, this was a major problem.

### What factors influence the context in which services operate?

There were many factors which were perceived to influence how services operated. These could be grouped into four areas: within the teams; the acute hospital; the community, and commissioning/management factors.

*Within-team factors* included the size and specialism of teams with smaller teams reported less resilience in being able to cope with staff absence due to ill-health or training. Larger teams reported issues with the balance between specialism or generalism – for example whether certain team members with particular skills should focus on specialist areas or whether all team members should cover all areas.

*Within the hospital*, teams were influenced by the size or make-up of the acute hospital - for example if the throughput of the ED was particularly busy or there were particular specialist units, or different referral patterns from different wards. Pressures on the acute hospital influenced delivery of care – for example patients being discharged before being reviewed by liaison services or patients with delirium being moved from ward to ward, adding to their confusion.

*Within the community*, the availability of primary care services, community mental health services and social care and support services, were major factors which influenced the safe and timely discharge of patients.

*Commissioners and service managers* had considerable influence over how services operated. This could include prioritising response times and setting targets for reducing re-admissions and length of stay. While being seen more quickly or spending less time in hospital is generally a sign of good clinical care, the target setting created an artificial focus on this particular measure, resulting at times in patients being discharged too early. In certain cases, managers gave instructions for LMHS not to see certain groups of patients, as commissioning had not been agreed (e.g. patients in a maternity unit linked to an acute hospital, or people who lived in an area that the commissioning team did not cover). Such decisions caused serious concerns for liaison staff about their professional obligations and duty of care to patients; and irritation and frustration in acute hospital staff, who had no other obvious recourse for help.

### Developing a programme theory for LMHS

It was apparent from the survey findings and liaison staff interviews that there were a variety of levels of complexity inherent in the way liaison services operated [39] and key trade-offs that affected activity and team performance. We identified 8 dimensions or levels of complexity:

- Liaison service configuration: We identified four patterns of liaison service operating in acute hospitals but recognized much diversity within this overall picture.

b) Networking of the service: all liaison services had horizontal and vertical partnerships. Within the hospital there were horizontal partnerships with a variety of acute hospital teams, and secondary mental health services including acute on-call teams. There were vertical links with primary care mental and physical health services, third sector organisations and social services.

c) Temporal instability: there were both expectations of short term and long-term changes.

d) Acute hospital configuration: there were organizational changes within acute hospitals which were relevant to liaison services and impacted on outcomes but were difficult to identify as within the scope of the service itself. These related, for example, to changing patterns of service delivery or specific short or long-term policy imperatives, including introduction of new teams within the acute hospital to aid discharge, or closure of nearby ED units resulting in an increase in attendances at other units.

e) Multiple interested parties: many actors have an interest in how liaison services work, they include patients, family or friends, liaison services themselves, acute hospital staff and managers, mental health managers, commissioners for physical health services, mental health service commissioners, community mental health services, GPs and primary care services, social care sector, third sector services, psychological services and acute mental health on-call teams.

f) Clinical focus: there are no agreed criteria for referral to liaison services or shared understanding between liaison staff and acute hospital staff of who should be referred. Mental ill health can be diagnosed using well recognised diagnostic criteria, but depression in the context of physical illness is not so straightforward, for example even mild sub-threshold symptoms of depression can have a deleterious effect on patient outcome and hospital costs.

g) Inherent clinical complexity of the work: mental ill health in the context of physical ill health has multiple different forms and contested causes. There is a synergistic interplay between mental health and physical illness which cannot be captured by a simple concept of co-morbidity.
h) The impacts of a liaison service could not be captured by a single logic model as described by NHS England. For example, processes that result in an improvement in response times are different to those involved in treating mental disorder in patients referred to services and may even be in conflict with each other. The nature of LMHS is likely to be best described in a number of logic models, organised according to key features such as outcomes – for example the logic model for a services’ organisational outcomes is likely to be different from that for clinical outcomes.

Against this background of complexity, the research team identified 6 trade-offs or tensions within services that affected how they operated. These were:

- Intensity of work with individuals versus the numbers of referrals that could be seen.
- Acute or urgent work versus non-acute more complex work.
- Desire to take over the care of the patient versus be part of an integrated system.
- Use of evidence-based practice versus innovative care versus patient-centred care.
- Specialisation versus generalisation.
- Diversity of roles (working in several settings) versus continuity of roles (working in one or two settings only).

It was apparent from the range of different outcomes and objectives within and across liaison services that a single logic model could not be used to adequately capture the way liaison services effected change in outcomes.

As exemplars we developed two models linked to common organisational outcomes: response times and length of hospital stay. Response times are regarded by referrers to liaison services as a key performance indicator [40]. We focused on the planned developments in liaison services [1], particularly how an increase in staffing levels according to commissioning guidelines should impact on these targets [1]. We focused on organisational outcomes, as most liaison services collect, or access these data, for performance monitoring. Although improved patient outcomes are perceived as being important by staff and NHS England, collection of meaningful outcome data is challenging. Many patients are only seen for a single assessment and patients present with a wide range of differing mental and physical health problems. The Royal College of Psychiatrists has published a Framework for Recording Outcomes and Measurement in Liaison Psychiatry [41], which should improve consistency in data collection, but the framework has not been universally adopted by services. A bespoke liaison symptomatic outcome measure has also been developed recently [42] but is not in widespread use.

All our exemplars involved more than one pass’ through the process, with a recognition that any change would be dynamic, and would involve a balance of some of the trade-offs, described above.

#### A logic model to explain mechanisms involved in reducing response times for liaison services

**Fig. 1:**
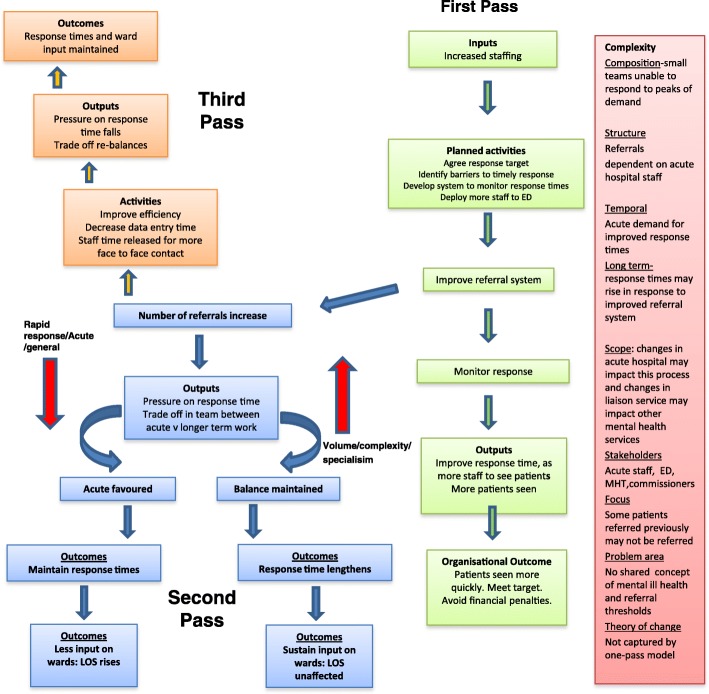
Three pass logic model to explain mechanisms and trade-offs involved in target of improved response times in ED for LMHS

Figure 1 shows the logic model we have developed to explain the impact an increase in liaison staffing may have on response times. The model assumes that this increase in personnel is occurring in the context of current developments in liaison services and is supported locally by the relevant commissioners.

The boxes in green show the first pass through the model. Increased staffing levels enable the team to see more patients and to seem those patients more quickly. It also enables the liaison team to develop a system for monitoring response times, an improved referral system (e.g. electronic rather than paper) and a way of recording response times which can be fed back to the acute Trust and commissioners. Time is also available to provide education to hospital staff about the role of the liaison team and suitability of referrals. The response times are monitored with an agreed suitable threshold (e.g. within one hour of referral for ED referrals or within 24 h for non-acute ward referrals). As there are more staff in the team, the response times improve. This means the team meets its targets for commissioners, and the ED meets its 4 h wait targets for mental health. In this first pass model, the increased staffing leads directly to an improvement in response times. However, if the model is allowed to run, various other outcomes may occur and the tensions and trade-offs in the system become more apparent, as do the levels of complexity in which the model is running.

The boxes in blue show the second pass through the system. A focus on the referral system and education of acute hospital staff has resulted in improved referral guidelines, a streamlining of the referral process, and greater awareness of the liaison service by hospital staff. This has resulted in an increase in referrals to the liaison service due to a variety of different factors; it is easier to refer to liaison service via the on-line system, the service has a higher profile, the rapid response encourages referral as psychiatry referral is no longer seen as a potential delay, etc. This increase in referrals now produces a tension or trade- off between the rapidity of response and the number of patients who can be seen. There may also be tensions between the diversity and continuity of roles within the team. More staff may be required to switch from their usual role to the front line acute service to meet the extra demand. There will also be tensions between the balance of acute hospital work and the needs of patients with severe mental health issues who remain as in-patients in the acute hospital. If the trade-offs all move towards an acute focus, the response time target will continue to be met, but there will be potential losses or disruptions to care of other patients, with potential deleterious consequences on their clinical outcome. The alternative is that response times will fall which may incur financial penalties and disruption to the acute hospital.

The boxes in orange show a third pass through the model. This suggests that there may be other creative ways to manage an increase in referrals. One possibility is that the additional expansion of the team enables key staff to improve the data-recording and efficiency of the assessment process, so that part of the time spent recording data is released for patient-care. This means additional patients can be seen. Alternately, the increase in referrals could be managed by increasing the inputs to the model and negotiating with commissioners for more staff.

The levels of complexity, which are shown in the box in light red, suggest the model may differ according to various factors and that the theory of change is not captured by a one-pass model. These factors include the initial size and composition of the liaison team, the historical and future referral patterns of hospital staff, the focus on short term or longer term outcomes, changes in the acute hospital which impact on the model (there may be a reduction or increase in hospital beds), influences of a variety of potential stakeholders (e.g. acute hospital managers and commissioners), and the focus of patient care.

#### A logic model to explain mechanisms involved in reducing length of stay

**Fig. 2:**
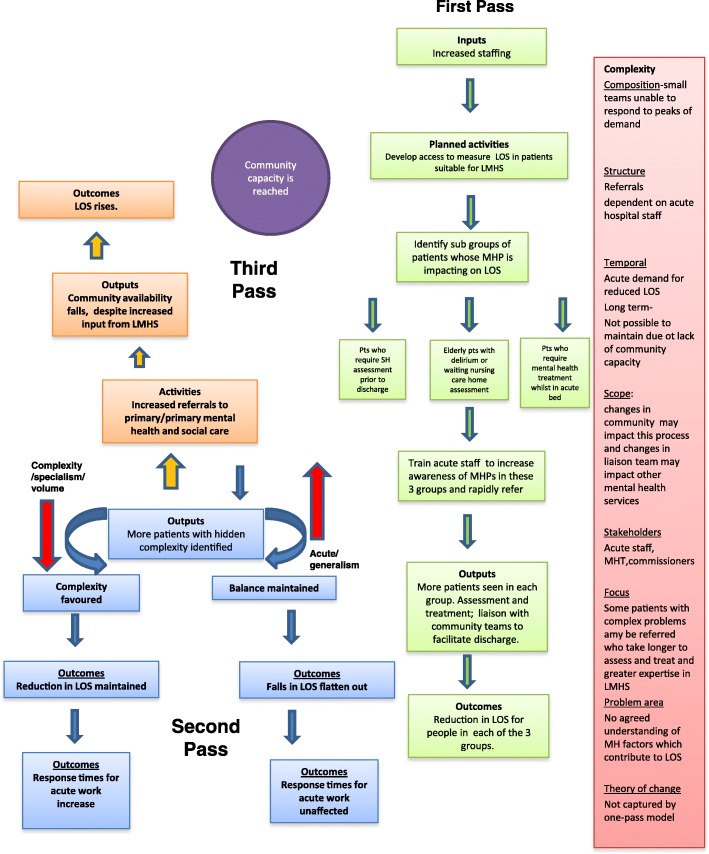
Three pass logic model to explain mechanisms and trade-offs involved in target of improved length of stay (LOS) for patients with mental health problems (MHP) admitted as an emergency

Figure 2 shows a similar model for length of stay. The green boxes again show the first pass through the system. As staffing increases, the LMHS are able to work with the acute hospital to identify sub-groups of patients with mental health problems who have longer than expected lengths of stay: patients who have self-harmed who required assessment and management from LMHS prior to discharge; patients with delirium/dementia who are waiting a nursing care home assessment; and patients who require treatment for their mental health problems whilst in an acute bed (e.g. patient who is psychotic but has developed cardiomyopathy secondary to clozapine). Ward managers on the main medical and surgical wards are made aware of these patient groups, through additional training and support to improve identification. More patients with these problems are seen by LMHS who have particular specialised skills in these areas. More patients are assessed, treated and referred to community services. Length of stay for these patients falls, and the LMHS meet a key performance target.

The blue boxes show the second pass through the system. More patients with complex problems are referred which puts pressure on the specialist staff in LMHS. This creates a tension between specialism and generalism and complexity versus acute work. More patients with complex patients can be seen, but at the expense of pressure on wait times in other areas of the service (e.g. ED).

The orange boxes show the third pass through the model. The increased referrals from LMHS to community services has filled what limited capacity there was for community support. Despite the work from the LMHS, the route out of hospital is severely impeded and length of stay begins to rise. There are many other ways the model could develop depending upon local availability of services, tensions in the team, priorities set by commissioners etc. The finite nature of the resources of mental health professionals may also impact upon the whole mental health system and is included as one of the complexities in ‘scope’ in the pink box. The increased staffing of the LMHS may denude staffing levels in other mental health teams, upon whom LMHS are dependent so that discharge of patients to the community can be expedited.

## Discussion

There are challenges for developing logic models to evaluate services or interventions with complex aspects [43]. Different outcomes have different mechanisms of action and it is overly simplistic to attempt to capture these different processes in one single, static model. One-pass models fail to represent the unintended changes in the system that occur following a significant change in service practice. This notion has been termed ‘emergence’ and is based on the principal that when a system changes, so does the behaviour of its agents, and the behaviour of the system as a whole [44]. An iterative approach may be required to fully capture the long term impact of change [45].

All models require a degree of contextual understanding and our work identified eight sources of complexity that may to a greater or lesser extent disrupt a simple theory of change. We also identified a series of tensions or trade-offs within services that can drive logic models in different directions, resulting in different outcomes. These trade-offs are reflected in day-to-day clinical practice within services and create challenges for delivering fully integrated liaison services, as we will report elsewhere [Keeble J, Walker A^,^ Guthrie E^,^ Trigwell P^,^ Quirk A^,^ Hewison J, House A. Integrated liaison psychiatry services in England: a qualitative study of the views of liaison practitioners and acute hospital staffs from four distinctly different kinds of liaison service. Submitted to BMC Health Services Research].

Recent debates about the nature of LMHS have not engaged with this degree of complexity. For example, the policy of making a structural intervention (increasing numbers of certain staff to so-called CORE-24 levels) to allow a 24/7 rapid response to ED referrals, does not accommodate either the emergent effects of such a policy or the opportunity cost of the trade-offs necessary to allow its implementation. The same criticism applies to proposals to make reduced length of inpatient stay the driving force behind service provision, resourced by teams where lack of specialisation within the team is almost a sine qua non.

Theories of change in relation to LMHS are relatively under-developed, and this paper represents an early attempt to delineate potential mechanisms for key outcomes which have been prioritised by government. The nascent models presented are derived from cross-sectional work and will need to be refined and developed further, with the addition of service user perspectives. These models focus on organisational outcomes, but liaison services also provide other key services to the acute hospital, including treatment and support for patients, education and support for staff and education for patients and their significant others. As programme theory develops in this area, these other more subtle but important functions of LMHS will need to be captured.

One of the important functions of logic models is to provide a vehicle for theory-building as a basis for understanding common mechanisms necessary for change [45]. The current expansion in liaison services across England provides an opportunity for service expansion to be studied prospectively [46–49], as part of which more detailed mixed methods, rigorous evaluations can be undertaken.

Our findings are important for commissioners of liaison services. Investment in the NHS should be subject to scrutiny and monitoring, but a reliance on simple target setting may result in services that are unbalanced and not patient-centred. Targets may need to be reviewed on a regular basis, together with other data that reflect the wider impact of the service, and any external changes in the system that affect performance (such as changes in primary care or social care).

## Conclusions

The strengths of this study include a survey of all 168 liaison services in acute hospitals in England, data from the largest qualitative study of liaison staff in England, chosen from services that are representative of the four different types of liaison teams currently in operation in the country.

Limitations of this study include the absence of patients’ experiences and views of liaison services. A further aspect of the programme of the research (LP-MAESTRO) will focus upon this important area, which will further inform model development in the liaison mental health field.

In addition, our findings are specific to liaison services in England and may not be generalisable to liaison services in other countries or liaison services that exist in non-acute hospital Trusts (e.g. cancer hospitals). Although programme theories by nature are portable in that the context and outcome may change but the mechanism of action may remain the same, health systems that are funded differently (e.g. tariff based systems, private insurance systems) may require different models.

## Abbreviations

ED: Emergency Department
LMHS: Liaison Mental Health Services
LP-MAESTRO: Liaison Psychiatry: Measurement and Evaluation of Service Types, Referral Patterns and Outcomes
MHA: Mental Health Act
